# Differences in the morphology, physiology and gene expression of honey bee queens and workers reared *in vitro* versus *in situ*

**DOI:** 10.1242/bio.036616

**Published:** 2018-10-25

**Authors:** Daiana A. De Souza, Osman Kaftanoglu, David De Jong, Robert E. Page, Gro V. Amdam, Ying Wang

**Affiliations:** 1 Faculdade de Medicina de Ribeirão Preto, Universidade de São Paulo, Ribeirão Preto, São Paulo 14049-900, Brazil; 2 Department of Entomology and Plant Pathology, North Carolina State University, Raleigh, NC 27695-7613, USA; 3 School of Life Sciences, Arizona State University, Tempe, AZ 85287-4501, USA; 4 Department of Entomology and Nematology, University of California Davis, Davis, CA 95616, USA; 5 Faculty of Environmental Sciences and Natural Resource Management, Norwegian University of Life Sciences, Aas 1432 Ås, Norway

**Keywords:** Honey bee queen quality, Geometric morphometrics, Carbohydrate metabolism, Ovariole number, Caste development

## Abstract

The effect of larval nutrition on female fertility in honey bees is a focus for both scientific studies and for practical applications in beekeeping. In general, morphological traits are standards for classifying queens and workers and for evaluating their quality. In recent years, *in vitro* rearing techniques have been improved and used in many studies; they can produce queen-like and worker-like bees. Here, we questioned whether queens and workers reared *in vitro* are the same as queens and workers reared in a natural hive environment. We reared workers and queens both *in vitro* and naturally in beehives to test how these different environments affect metabolic physiology and candidate genes in newly emerged queens and workers. We found that sugar (glucose and trehalose) levels differed between queens and workers in both *in vitro* and in-hive-reared bees. The *in vitro*-reared bees had significantly higher levels of lipids in the abdomen. Moreover, hive reared queens had almost 20 times higher levels of vitellogenin than *in vitro*-reared queens, despite similar morphologies. In addition, hive-reared bees had significantly higher levels of expression of *mrjp1*. In conclusion, *in vitro* rearing produces queens and workers that differ from those reared in the hive environment at physiological and gene expression levels.

This article has an associated First Person interview with the first author of the paper.

## Abstract (precis)

**Summary:** We used *in vitro*-rearing methodology to investigate how differences in rearing conditions, including nutrition, affect the morphology, metabolic physiology and gene expression of honey bee queens and workers.

## INTRODUCTION

Female honey bee queens and workers differ greatly in many phenotypic aspects, which are in turn related to their social roles. Queens are larger (150–250 mg), with a huge abdomen that has well-developed reproductive apparatuses: ovaries with 200–400 ovarian filaments (ovarioles) and a spermatheca (Snodgrass, 1956), which makes her able to perform one of her most important social tasks, laying eggs. Workers are smaller (80–110 mg) and have specialized morphological traits, such as hypopharyngeal glands, wax glands, notched mandibles and pollen baskets, corresponding to their social roles, including nursing brood, constructing the nest and foraging for nectar and pollen. Workers also have ovaries, but their size is much reduced compared to those of the queen (they have a single pair of ovaries, each with less than 20 ovarioles). Workers usually do not produce eggs when the queen is present in the colony (Ruttner, 1983; Graham, 1992).

Honey bee queens and workers develop from the same diploid eggs; their distinct phenotypes are determined by different quantities (Lindauer, 1952) and quality (Haydak, 1970) of food that female larvae receive from adult worker (nurse) bees (Michener, 1974; Page and Peng, 2001). During the first three days of larval life, queen- and worker-destined larvae are fed with a similar quality and quantity of royal jelly, which is secreted by hypopharyngeal glands and mandibular glands of young adult workers. Thus, larvae at this stage are bipotential and can develop into queens or workers. After the third larval instar, queen-destined larvae (in queen cells) and worker-destined larvae (in worker cells) are differentially fed; queen larvae continue to receive large amounts of high quality food (royal jelly), while the worker larvae food is nutritionally inferior worker jelly (Dietz and Haydak, 1971; Winston, 1987; Graham, 1992). By grafting worker larvae into queen cells, beekeepers can raise typical queens if grafting is done no later than the third instar. If older larvae are grafted, such as at the fourth instar, the resulting queen will have a reduced body size, fewer ovarioles and a smaller spermatheca (Weaver, 1957; Woyke, 1971; Dedej et al., 1998; Tarpy et al., 2011). Nutritional variation in worker larvae also affects their phenotypes. The food restriction at the end of the worker larval stage, compared to what is fed queen-destined larvae, results in fewer ovarioles per ovary and smaller individuals (Wang and Li-Byarlay, 2015). Thus, developmental divergence between a queen and a worker starts around the third instar of the larval stage and proceeds in a continuous process until eclosion.

The quality of a queen is highly associated with her reproductive ability, which is very critical for colony quality, including colony size, honey production, disease resistance and overwintering ability. Ovary size (ovariole number) is usually an indicator of queen reproductive capability and is generally associated with other phenotypic traits, such as spermatheca and body size of newly emerged queens (Hoopingarner and Farrar, 1959; Woyke, 1967, 1971; Kerr et al., 1970; Corbella and Gonçalves, 1982; Dedej et al., 1998; Kahya et al., 2008; Tarpy et al., 2011; De Souza et al., 2013; Rangel et al., 2013). External phenotypic traits, especially body size, are widely used by beekeepers for evaluating queen quality.

In recent years, *in vitro* feeding has been developed and used to raise queen-like bees and worker-like bees in a lab environment. This technique has great potential for honey bee research (i.e. it can be used in developmental biology studies) in controlled environments. Publicly available *in vitro* protocols can produce both queen-like and worker-like morphological phenotypes (Patel et al., 2007; Mutti et al., 2011; Kaftanoglu et al., 2011; Crailsheim et al., 2013). However, the absence of social control (feeding by nurse bees) and the artificial environment could impact on important traits, affecting the viability and physiology of these bees (Linksvayer et al., 2011; Leimar et al., 2012; De Souza et al., 2015). In fact, bees reared *in vitro* show great morphological plasticity; different feeding regimes can produce queen-like, worker-like or intercaste phenotypes. Intercastes have mixed morphological traits of queens and workers, such as body size, mandible shape, hind leg characteristics, ovariole number, and absence or presence of a spermatheca (De Souza et al., 2015). Frequently, the body mass versus ovariole number ratio is dissociated. In addition, we repeatedly observed that *in vitro* queen-like bees were larger than natural queens, visually presenting more fat in their abdomens (Y.W., unpublished). This complex phenotypic variation found in *in vitro* feeding studies lead us to question the degree of similarity between queen-like bees and worker-like bees produced by *in vitro* feeding compared to naturally-reared queens and workers.

Here we investigated whether morphological features of female phenotypes (queens and workers) reared in a colony differ from those of bees reared *in vitro*. We also investigated whether these morphological differences affect physiological traits that are important for their social roles. First, we measured the blood sugar (glucose and trehalose) levels in these bees, as well as abdominal lipid reserves; these are indicators of metabolic states in newly emerged bees. Then, we looked at genes that have to do with reproduction and nutritional status: vitellogenin (*vg*) and major royal jelly protein 1 (*mrjp1*). Vitellogenin has a direct impact on ovarian activation and egg production in queens. Additionally, vitellogenin (Vg) regulates the transition of workers from nurses to foragers; it is also a component of the larval food produced by workers (Engels et al., 1990; Amdam and Omholt, 2002; Guidugli et al., 2005; Seehuus et al., 2006; Cruz-Landim, 2009). MRJP1 is a major component in royal jelly and has a significant influence on honey bee development and ovarian physiology (Wegener et al., 2009; Kamakura, 2011). This makes mrjp1 and vg useful markers for comparing *in vitro*-reared and naturally produced queens and workers.

## RESULTS

### Identification of *in vitro* queen-like and worker-like bees

**Fig. 1.**
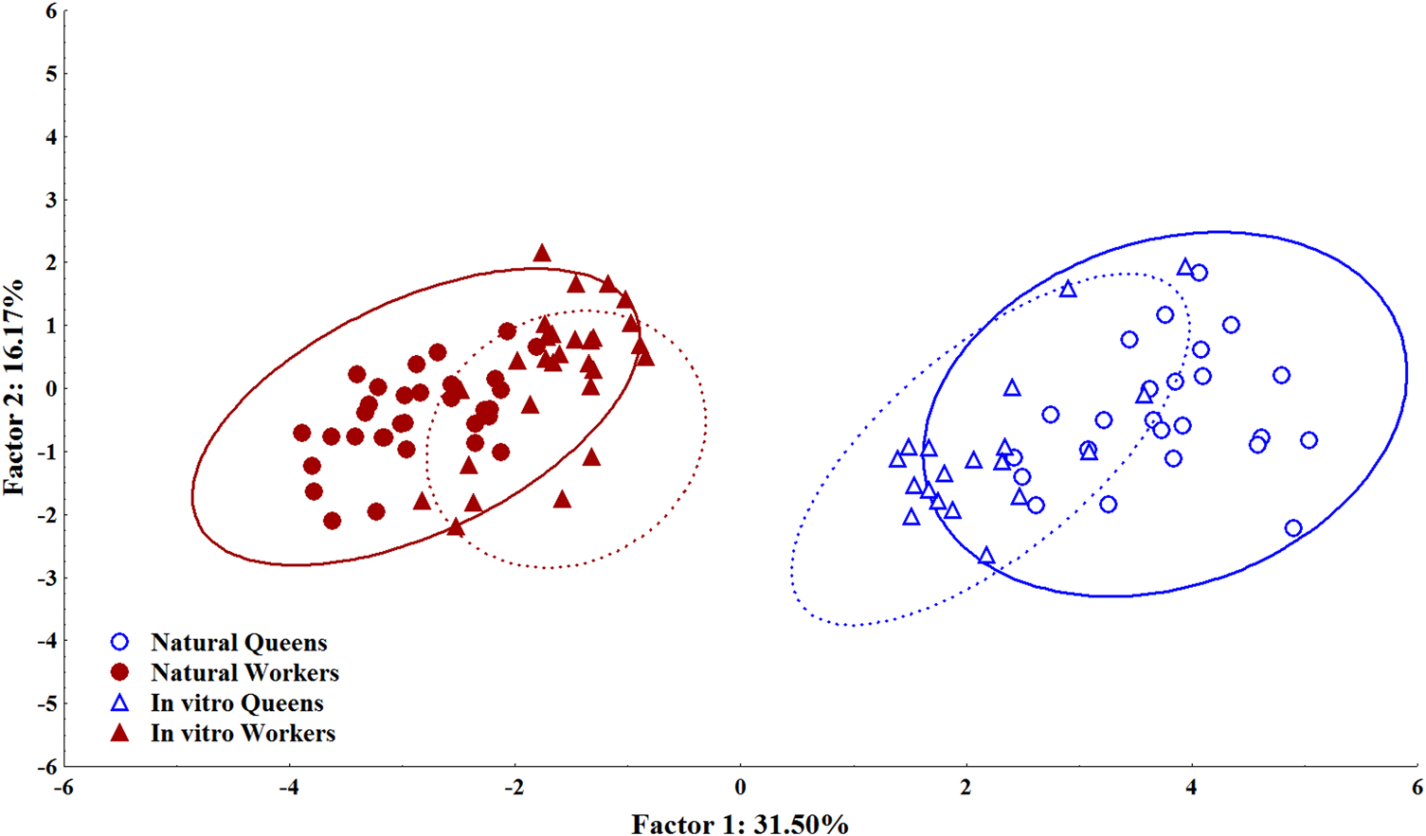
**PC1 and PC2 of a principal component analysis (PCA) of multiple morphological traits of *Apis mellifera*.** There is a clear separation of the bees among the caste groups both in hive-reared and in *in vitro* reared bees. Ellipses enclose each of the caste groups; a continuous line includes the hive-reared and a dotted line the *in vitro*-reared phenotype groups.

The *in vitro* reared queen-like and worker-like bees were identified based on a comparison of the features of hive-reared queens and workers (Fig. 1). Principal component analysis (PCA) revealed that 31.50% of the total variation of morphological structures can be explained by the first principal component (PC1), and 16.17% of the remaining variation can be explained by the second principal component (PC2). The eigenvalue of PC1 was 5.039577 and that of PC2 was 2.5869456 (Table S1). Using these grouping results of naturally reared samples, the centroid distance of these groups was taken as a reference and *in vitro* bees were classified and selected for further assays. Samples of *in vitro* raised bees that emerged with intermediate morphology, currently known as intercastes (De Sousa et al., 2015), were discarded from our sampling and the analyses described below were performed only with the bees that emerged with clearly adult queen-like or worker-like phenotypes.

**Fig. 2.**
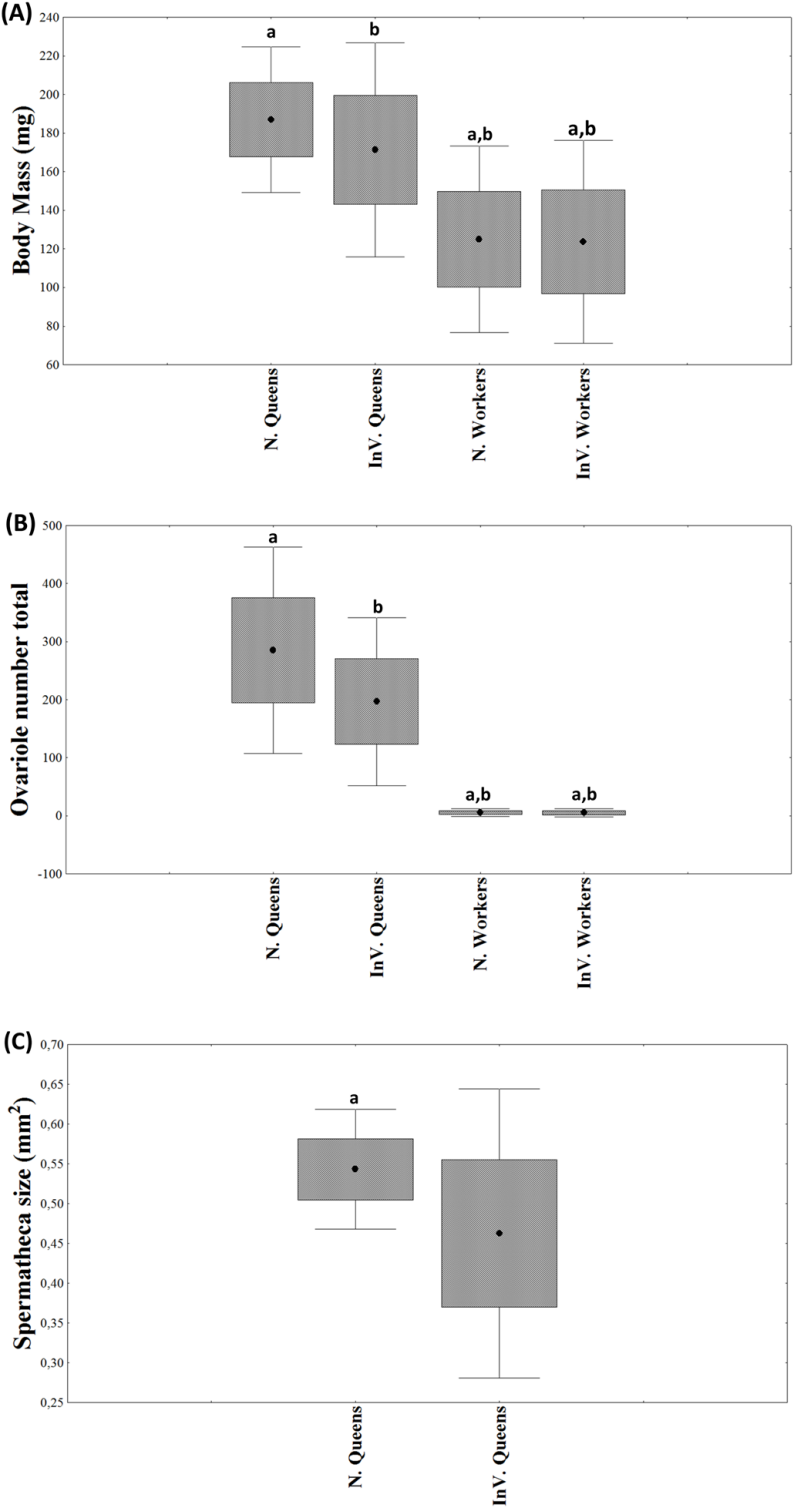
**Comparison of morphological characteristics of newly emerged honey bees that are hive reared (N.) or *in vitro* reared (In.V).** (A) Body mass (mg) (*n*=52-60). (B) Total number of ovarioles (*n*=20-35). (C) Spermatheca area (mm^2^) (*n*=25-30). Different letters (a–c) over the bars indicate significant differences among the groups. (Mean±s.e., *P*<0.05; Factorial-ANOVA).

Queens and workers produced in both hive and *in vitro* rearing environments significantly differed in emergence weight (Fig. 2A). The rearing environment (*in vitro* rearing versus hive rearing) significantly affected honey bee weights (*F*_(1,56)_=26.39, *P*≤0.001). The *in vitro* reared queen-like bees were significantly smaller than hive-reared queens (174.9±24.5 versus 187.0±18.8 mg; *F*_(1,23)_=6.67, *P*=0.01); in contrast the worker-like bees did not differ between naturally and *in vitro* reared bees (124.9±24 versus 123.6±11.9 mg, respectively; *F*_(1,142)_=1.000 *P*=0.32). The interaction effect between the caste phenotype and the environment in which the bees were reared was not significant (*F*_(1,56)_=3.38, *P*=0.06), indicating that the effect on the weight at adult emergence was independent of the environment in which they were reared. *Post hoc* analysis further supported the conclusion that queens reared *in vitro* are lower in weight at adult emergence compared to naturally-reared queens (Fisher LSD: *P*=0.0495) but this was not observed among workers (Fisher LSD: *P*=0.8962, Fig. 2A).

The ovariole number also differed significantly between hive reared and *in vitro* reared queens (285.0±90.7 versus 196.8±73.8, respectively; *F*_(1,20)_=12.16, *P*=0.002). The hive-reared queens had larger ovaries. There was no significant difference in ovariole number between hive workers and *in vitro* workers (5.6±3.3 versus 5.2±3.6, respectively; *F*_(1,30)_=0.41, *P*=0.52; Fig. 2B). There was a significant interaction effect between the caste phenotype and the environment in which the bees were reared (*F*_(1,30)_=18.38, *P*≤0.001), indicating that ovariole number at emergence was affected by the environment in which they were reared. *Post hoc* analysis further showed that the queens reared *in vitro* had fewer ovarioles compared to naturally-reared queens (Fisher LSD: *P*≤0.001), but this was not observed among workers (Fisher LSD: *P*=0.8962, Fig. 2B).

The hive-reared queens had significantly larger spermathecae than *in vitro*-reared queens (0.543±0.038 mm^2^ and 0.462±0.097 mm^2^, respectively; *F*_(1,12)_=7.47, *P*=0.01; Fig. 2C).

### Metabolic physiology

**Fig. 3.**
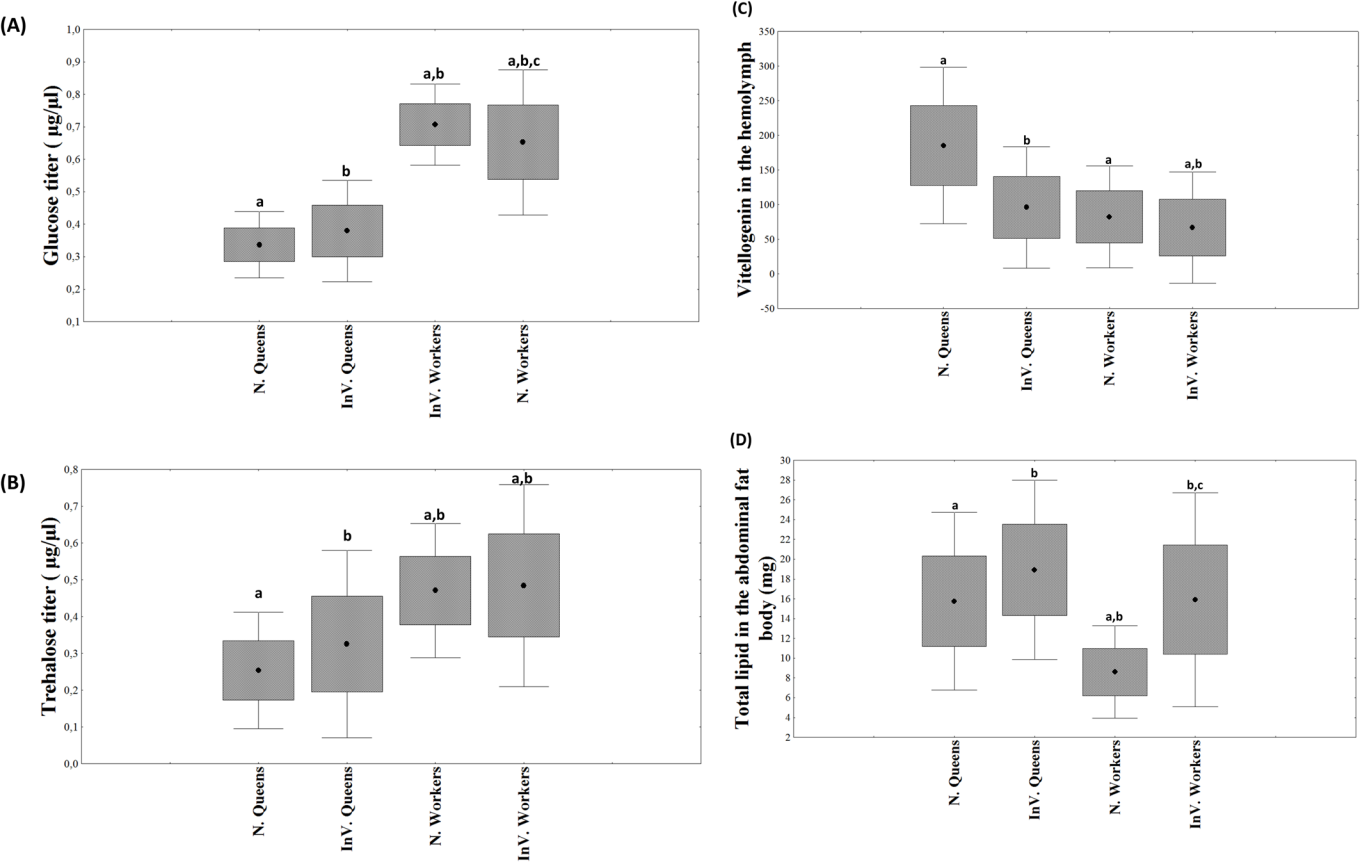
**Physiological parameters of newly emerged honey bees that are hive reared (N.) or *in vitro* reared (In.V)**. Hemolymph titers of (A) glucose (*n*=20), (B) trehalose (*n*=20) and (C) vitellogenin (*n*=15–20) and (D) lipids in the abdominal fat body (*n*=19-24). Different letters (a–c) over the bars indicate significant differences among the groups. (Mean±s.e., *P*<0.05; Factorial-ANOVA).

The groups had significantly different glucose levels (*F*_(1,39)_=242.79, *P*<0.001) and trehalose levels (*F*_(1,39)_=62.19, *P*<0.001). Queens and workers significantly differed in blood glucose and trehalose titers (*F*_(1,76)_=316.05, *P*≤0.001; *F*_(1,19)_=54.74, *P*≤0.001, respectively). The queens had much lower levels of both glucose and trehalose in their blood. There was a significant interaction effect between the caste phenotype and the environment in which the bees were reared in the comparison of glucose levels (*F*_(1,76)_=7.22, *P*=0.008), indicating that the glucose levels were dependent on the environment in which they were reared, especially among the workers. *Post hoc* analysis further showed that the workers reared *in vitro* had lower glucose levels compared to naturally-reared workers (Fisher LSD: *P*=0.0353), but this was not observed among queens (Fisher LSD: *P*=0.1014, Fig. 3A). When we examined trehalose levels, there was no significant interaction effect between the caste phenotype and the environment (*F*_(1,56)_=1.29, *P*=0.25, Fig. 3B), indicating that trehalose level differences observed among the caste phenotypes were independent of the environment in which they were reared. This difference in glucose and trehalose titers between naturally-reared queens and workers that was also found in *in vitro* reared queen-like and worker-like bees is consistent with the morphological differences that were used to classify the *in vitro*-reared bees.

The differences in Vg titer among the four groups were significant (*F*_(1,40)_=21.49, *P*<0.001). The level of Vg in the hemolymph was significantly higher in hive-reared queen bees compared to all other groups. There was no difference among natural workers, *in vitro* queens and *in vitro* workers (Fig. 3C). This indicates that the *in vitro*-reared queen-like bees had much less Vg, even though they were fed *ad libitum*, when compared to hive-reared queens. These results also confirmed that workers have less Vg than queens. There was a significant interaction effect between the caste phenotype and the environment in which the bees were reared (*F*_(1,79)_=13.28, *P*=0.0004), indicating that the Vg levels of each caste were dependent on the environment in which they were reared, especially among the queens. *Post hoc* analysis further showed that the queens reared *in vitro* had lower Vg levels compared to naturally reared queens (Fisher LSD: *P*<0.001) but this was not observed among workers (Fisher LSD: *P*=0.277, Fig. 3C).

The differences in abdominal fat among the four groups were also significant. Both hive-reared and *in vitro*-reared queens had much more abdominal fat than workers (*F*_(1,37)_=23.86, *P*≤0.001). Queens reared in the hive versus *in vitro* did differ in lipid titers (*F*_(1,17)_=3.63, *P*=0.02); the same was found among workers, (*F*_(1,18)_=26.40, *P*<0.001); *in vitro*-reared bees had significantly more lipids than hive-reared bees. There was a significant interaction effect between caste phenotype and the environment in which the bees were reared (*F*_(1,74)_=4.35, *P*=0.04), indicating that the *in vitro* nutritional environment significantly affected the levels of abdominal fat in both castes. *Post hoc* analysis further supported the observed impact of the *in vitro* environment on fat levels (Fisher LSD_queens_: *P*=0.02; Fisher LSD_workers_: *P*<0.001; Fig. 3D)

### Gene expression

**Fig. 4.**
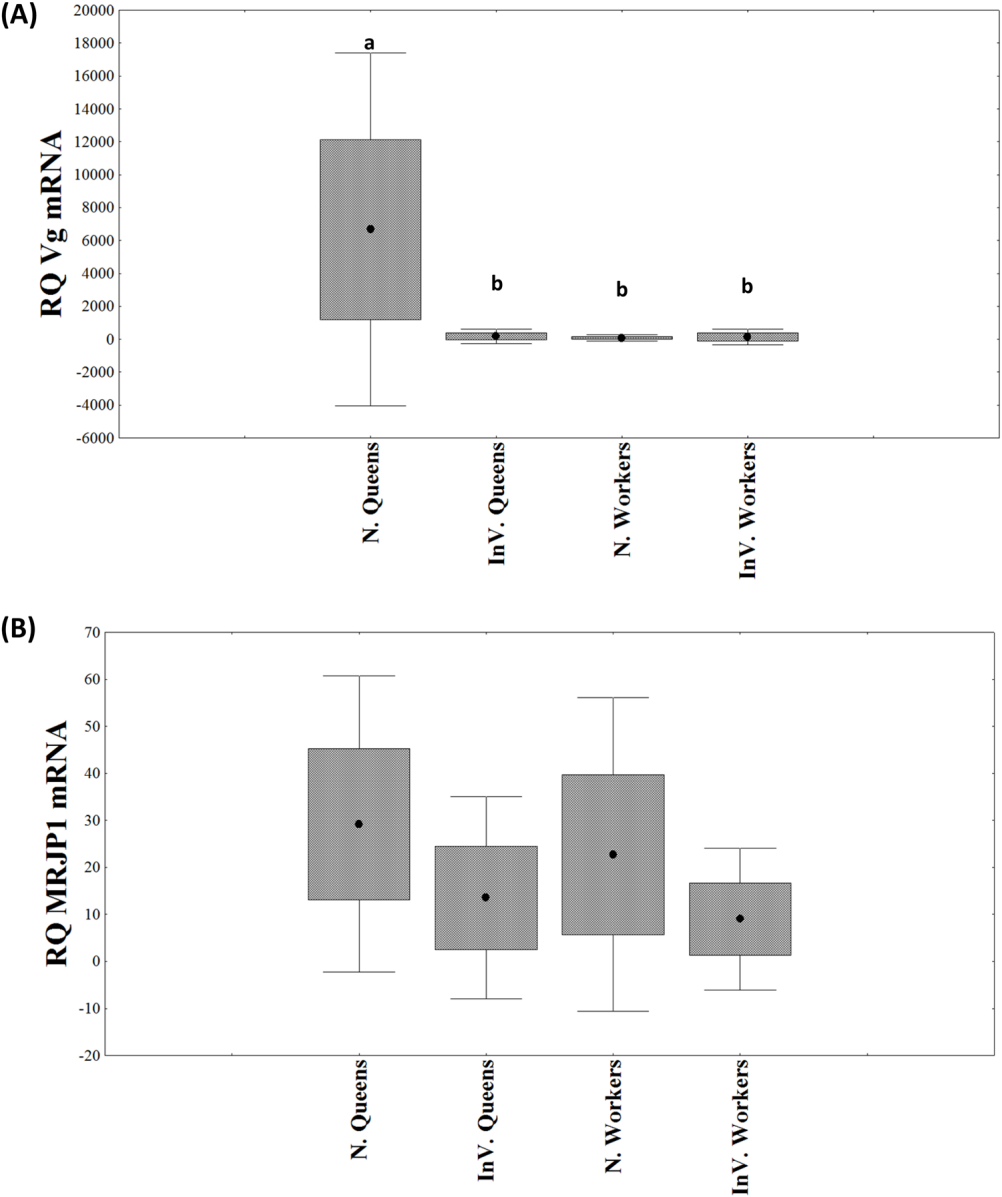
**Candidate gene expression in newly emerged honey bees that are hive reared (N.) or *in vitro* reared (In.V).** Relative expression of (A) *vg* in the fat body tissue (*n*=15), (B) *mrjp1* in the fat body tissue (*n*=15). Different letters (a–c) over the bars indicate significant differences among the groups. (Mean±s.e., *P*<0.05, Factorial-ANOVA).

*vg* and *mrjp1* expression were measured in the four treatment groups. The rearing environment significantly affected *vg* and *mrjp1* mRNA levels (vg: *F*_(1,26)_=11.48, *P*=0.002 and mrjp1 *F*_(1,22)_=14.10, *P*=0.001; Fig. 4).

*vg* gene expression in hive-reared queens was 19 times higher than in *in vitro* queens (*F*_(1,13)_=19.46, *P*<0.001), and 35 times higher than in workers from both rearing systems (*F*_(2,22)_=15.44, *P*=0.00006; Fig. 4A). However there was no difference in *vg* expression between hive-reared workers and *in vitro* reared bees (*F*_(1,11)_=0.40, *P*=0.53). *mrjp1* gene expression did not differ between hive-reared queens and workers (*F*_(1,8)_=0.16, *P*=0.69), nor between *in vitro* reared groups (*F*_(1,11)_=2.99, *P*=0.11). However, the hive-reared bees had greater expression of this gene than *in vitro*-reared bees (*F*_(1,22)_=14.10, *P*=0.001; Fig. 4B), which means the nutritional environment directly affected *mrjp1* expression.

## DISCUSSION

Our main goal was to determine whether and how much queen-like and worker-like bees reared on an artificial diet differ from natural queens and workers in terms of reproductive morphology, physiology and gene expression. We classified *in vitro* queens and workers as queen-like and worker-like bees based on a comparison with the external morphological characters of natural queens and workers, including size of the head and the shapes of their mandibles and pollen baskets, which are key morphological traits that differ between queens and workers. Then we evaluated the body weight, ovariole number and the size of the spermatheca among *in vitro* queens, *in vitro* workers, natural queens and natural workers. Based on our results, we conclude that *in vitro*-reared queen-like and worker-like bees that are characterized by their external morphological traits differ in body weight, ovariole number and the size of the spermatheca when compared to natural queens and workers. The morphological traits that we measured are commonly used in apicultural practice to distinguish workers from queens, and to assess queen quality. These results lead to a new series of questions about whether differences in these traits between queens and workers fully represent differences in queen and worker phenotype and whether these morphological traits reflect the quality of adult honey bee females.

Consistent with these morphological trait differences, glucose and trehalose titers in the hemolymph differed between queens and workers in both naturally and *in vitro*-reared bees. The queens had lower sugar titers than workers. This is the first time that caste-associated metabolic differences in adult bees have been identified. In many insect species, including honey bees, larvae are fed continuously until pupation; they accumulate energy reserves for use during the pupal and early adult phases. Since the fat body, the main organ for metabolism, is carried over from the larval stage to the newly emerged adult stage, metabolic patterns in the larval stage and the newly emerged adult stage can overlap to some degree. Therefore, we suggest that differences in carbohydrate metabolism between queen- and worker-destined larvae may carry over to newly emerged bees, even when exposed to the same nutritional environment, as in the *in vitro* rearing method adopted here. In fact, queen and worker larvae receive different quantities of sugar from nurse bees and they differentially express insulin peptides and insulin receptors, which are responsible for carbohydrate metabolism (Patel et al., 2007; de Azevedo and Hartfelder, 2008; Mutti et al., 2011; Wolschin et al., 2011). Although gene knockout of insulin peptides in female larvae does not change glucose and trehalose titers, it is still unclear whether the insulin pathway is involved in larval carbohydrate metabolism since the function of insulin receptors is unknown (Wang et al., 2013). Moreover, it is worth noting that the glucose and trehalose patterns we observed here are similar to what we found in a previous study. In that study, we used short-term starvation of fifth instar larvae and observed higher glucose and trehalose titers in the newly emerged bees (Wang et al., 2012b). In fact, worker larval development is associated with nutritional stress when compared to queen larval development. Therefore, further investigation on insulin and stress pathways may help us understand the mechanisms of queen and worker differentiation in honey bees.

All the *in vitro* reared larvae were grafted at the first instar and were fed *ad libitum* during the larval stage. Though most developed into queen and worker phenotypes, a great number of them became intercastes, as previously observed (De Souza et al., 2015). Clearly these differentially developed bees have different responses to the same nutrients during the larval stage. In this study, in order to increase genetic variation, we mixed larvae from three ‘wild-type’ sources, each of which had an open mated queen. Therefore, our results may reveal the effect of genotypic variation on queen and worker differentiation. However, it is remarkable that food manipulation by nurse bees can completely overwrite genotypic influences during larval development and completely control their phenotypes according to colony demands. On the other hand, we suggest from this study that artificial rearing is a useful way to examine phenotypic variation associated with nutritional variation and allows us to study the role of nutrition in honey bee development.

We found that hive-reared queens have much more vitellogenin, at both protein and mRNA levels, than hive workers and *in vitro*-reared bees (queens and workers). Vitellogenin is an essential storage protein for honey bees, with important functions in immune response, reproduction, and social behavior; it also affects honey bee health and lifespan (Amdam and Omholt, 2002; Amdam et al., 2004b, 2005). In general, natural queens have much higher Vg titers and *vg* mRNA than workers, which contributes to the high fertility and longer lifespan of queens. It is surprising that *in vitro* reared queens have very low levels of Vg and *vg* mRNA, even though they have the same morphological traits as natural queens. We also found a similar pattern in *mrjp1*, in which hive-reared queens had the highest *mrjp1* mRNA levels, especially compared to *in vitro*-reared queens. Normally, queen larvae are fed with protein-rich royal jelly, which contains approximately equal amounts of protein and carbohydrates (37.9% protein and 33.3% carbohydrate on a dry mass basis) (Hanser and Rembold, 1964; Rembold, 1973; Johannsmeier, 2001; Pirk et al., 2010). For *in vitro* rearing, the artificial diet usually contains 53% commercial royal jelly and more sugar in the composition to supplement the crystalized sugar in stored royal jelly. This potentially results in less protein for the larvae. Additionally, it has been reported that after a period of storage, royal jelly (even in −80°C freezers) major proteins gradually degrade (Yamaguchi et al., 2013). Therefore, even though the *in vitro*-reared larvae can consume artificial diet *ad libitum*, they may have less available and digestible proteins from the diet and consequently have lower protein synthesis. On the other hand, we did not find that lipid levels were negatively affected by the artificial rearing method. Instead, the *in vitro*-reared queens and workers had high lipid titers that were similar to the levels found in hive-reared queens. It is widely accepted that queen-destined larvae are fed with a diet containing a higher sugar concentration, which acts as a phagostimulant, increasing food consumption and growth rate, differentially triggering hormone release and consequently causing queen phenotypic differentiation (Asencot and Lensky, 1984, 1985, 1988; Page, 2013; Hartfelder et al., 2015). Since sugar concentration in the artificial diet is relatively high, it may result in a higher consumption rate in *in vitro*-reared bees, which increases lipid synthesis. Apparently, protein metabolism was affected differently.

Overall, by using artificial rearing methods, we found that morphological traits cannot be fully translated into bee quality. Protein levels, especially Vg in newly emerged bees, are more sensitive to larval nutrition. Our study indicates that even though we can produce larger queen-like and worker-like bees in an artificial environment, the quality of the bees is lower than that of naturally-reared queens. However, on the other hand, the artificial rearing method provides a tool to dissect the developmental program in honey bees, to determine how it is affected by nutrition. Moreover, our physiological and gene expression data suggest that protein, lipid and glucose metabolisms are regulated independently during the honey bee larval stage.

## MATERIALS AND METHODS

### Samples

Three ‘wild-type’ (unselected commercial stock) colonies were used in this study. They were maintained at the Honey Bee Research Facility, School of Life Sciences, Arizona State University, Mesa, Arizona. Queens were confined to a fully drawn comb in an excluder cage (46×24×6 cm), according to Peng et al. (1992).

### Honey bee larvae reared *in vitro* and in the hive

The *in vitro* reared bees were produced based on an established protocol (Kaftanoglu et al., 2011). This protocol was chosen, after previous tests, as it provided the highest percentage of target phenotypes. Female larvae less than 24 h old (obtained by caging the queen on brood comb) were grafted directly onto the surface of abundant artificial food (53% royal jelly and 46% aqueous solution containing 6% D-glucose, 6% fructose, and 1% yeast extract) in Petri dishes. Healthy larvae were carefully transferred daily to a new Petri dish with new fresh food. The food was provided *ad libitum*, to all *in vitro* reared larvae. Larvae were kept in an incubator at 34°C and 80% RH until the defecation stage. Then, the larvae were transferred to a piece of filter paper and kept in an incubator at 34°C and 70% relative humidity until emergence as adults (Linksvayer et al., 2011). All *in vitro* reared bees were exposed to the same environmental and diet conditions throughout their ontogenic development.

The same batch of larvae from the same combs used for *in vitro* rearing was also used for rearing natural queens and workers. One-day-old larvae that were randomly selected for rearing natural queens were grafted to artificial queen cups and introduced into strong queenless colonies (Laidlaw, 1979). The remaining larvae in the comb were returned to the queenright colonies, where they developed into adult workers. The frames with hive-reared queen cells and worker brood were transferred to an incubator shortly before emergence. Subsequently, the newly emerged workers and queens were collected for analysis. Thus, the natural queens and workers had the same genetic makeup and ages as the bees reared *in vitro*. These naturally-reared queens and workers served as controls for phenotypic classification of the artificially reared bees.

### Caste phenotyping

Phenotyping was performed using geometric-morphometric characterization of honey bee castes following the De Souza et al. (2015) protocol. This technique is based on characterization of various phenotypic traits, including head, mandible, metatarsus, body mass and ovariole number of newly emerged queens and workers. Using the phenotypic data from hive-reared queens and workers, we performed a principal component analysis (PCA) to separate queens and workers into two groups, then we classified *in vitro*-reared bees by adding their phenotypic data to the analysis. This allowed the *in vitro*-reared queens and workers to be classified based on the criteria that were established by the data from hive-reared queens and workers, taking the centroid distance from natural queens and workers as a reference distance point, with 98% confidence, as described in De Souza et al. (2015).

### Spermatheca and ovariole number measures

Spermathecae were dissected from the abdomens, fixed on a glass slide, photographed with a digital camera attached to a stereomicroscope (using a constant magnification) and evaluated using ImageJ software. The worker ovaries were dissected and counted with a stereomicroscope. The queen ovaries were postfixed in 3.7% neutral buffered formaldehyde for 24 h, dehydrated in increasing ethanol concentrations (in a sequence from 80, 90, 95%, 100%), treated with xylene, paraffin-embedded and sectioned, the ovary sections were stained with Hematoxylin-Eosin. The ovary slides were photographed under a stereomicroscope and the ovariole number was counted with ImageJ software. The magnification (sufficient to nearly fill the microscope field of view) was held constant for image analysis.

### Blood sugar and vitellogenin protein level

Two sets of hemolymph samples were collected from the abdomen of each bee to determine sugar (glucose and trehalose) and vitellogenin (Vg) levels. Glucose and trehalose titers were measured using enzymatic reactions (Hartfelder et al., 2013; Wang et al., 2013). 1000 µl of glucose reagent (Sigma-Aldrich) was added to 1 µl hemolymph and the solution was incubated at room temperature for 15 min. 100 µl of this solution was transferred to each well in microplates; each sample had three replicates. Eight glucose standards (0, 0.5, 1, 5, 10, 30, 50, and 100 mg/ml) were included in each microplate to produce a standard curve. Then absorbance at 340 nm was measured using a spectrophotometer (Bio-Rad xMARK Microplate spectrophotometer); the readings were translated to glucose concentration by comparison with a standard curve. Afterwards, 0.5 µl trehalase (Sigma-Aldrich) was added to each well in the same microplates and the solution was incubated at 37°C overnight and absorbance at 340 nm was measured again after the trehalose was broken down into glucose. The amount of trehalose was calculated using an equation: trehalose (mg) = glucose (mg)×342.3/ (180.2×2). Three replicates were tested for each sample. Final concentrations were determined by comparison with standard curves.

The concentration of vitellogenin in the hemolymph was estimated using SDS-PAGE (7.5% gel) of 1 µl samples of hemolymph, stained with Coomassie Brilliant Blue. The gels were scanned, and the images analyzed with ImageJ (http://rsbweb.nih.gov/ij/) software to quantify staining intensity of the vitellogenin bands, using apolipoprotein-1 for normalization of Vg levels (Guidugli et al., 2005).

### Abdominal lipid levels and gene expression

A set of abdominal fat bodies was collected from both *in vitro*- and hive-reared queens and workers and fresh-frozen in liquid nitrogen. For lipid level analysis, each abdomen, without digestive tract and sting apparatus, was homogenized in a 2:1 chloroform: methanol solution and dried down to a final volume of 2 ml. A lipid assay was performed using 1 ml of each sample, following the protocol of Wang et al. (2012a). Absorbance at 525 nm was measured and readings were converted to mg using a standard curve generated from cholesterol standards (Wang et al., 2012a). Three replicates were performed for each sample.

Another set of abdominal fat bodies was collected and flash-frozen in liquid nitrogen for gene expression analysis. RNA was extracted from both the *in vitro*- and hive-reared queens and workers using TRIzol Reagent (Invitrogen) (Wang et al., 2009). DNase (Qiagen) treatment was used to remove residual genomic DNA. The cDNA was synthesized using TaqMan Reverse Transcription Reagents (Applied Biosystems). Fifteen samples from each group were used for gene expression analysis. Two-step qRT-PCR (real-time) was performed on the samples with technical triplicates using ABI Prism 7500 (Applied Biosystems). Gene expression of vitellogenin (*vg*) (Amdam et al., 2003) and major royal jelly protein 1 (*mrjp1*) (Wang et al., 2012b) was measured using the ΔΔCt method (Pfaffl, 2001; Wang et al., 2009). The *actin* gene (GenBank: XM_623378) was used as the reference gene as it is one of the most stably expressed housekeeping genes in both honey bee larvae and adults, and in various honey bee tissues (Lourenço et al., 2008; Scharlaken et al., 2008); it is commonly used in gene expression studies in honey bees (Amdam et al., 2004a; de Azevedo and Hartfelder, 2008).

### Statistical analysis

The data was tested for normality based on Bartlett and Levene's homogeneity test and log transformed to approximate normality when necessary. A factorial ANOVA was used to test the effect of the treatments (*in vitro* versus hive-rearing methods) and the adult phenotype (queens versus workers) on reproductive structures (ovariole number and spermatheca), metabolic physiology (glucose, trehalose and lipid) levels, and the level of Vg protein and expression of vg and mrjp1 genes. All analyses were performed using STATISTICA 7.0 (StatSoft) software.

## Supplementary Material

Supplementary information
