# Supplementary material for: Differences in the morphology, physiology and gene expression of honey bee queens and workers reared in vitro versus in situ
Source: Biol Open. 2018 Oct 25;7(11):bio036616. doi: 10.1242/bio.036616 (PMC6262861; doi:10.1242/bio.036616)
Supplement: Supplementary information [file biolopen-7-036616-s1.pdf]

Table S1: Eigenvalues of the correlation matrix, and related statistics

	Eigenvalue	% Total variance
**Factor 1**	5.039577	31.49736
**Factor 2**	2.586946	16.16841
**Factor 3**	1.601537	10.00961
**Factor 4**	1.231425	7.69641
**Factor 5**	1.037487	6.48430
**Factor 6**	0.847643	5.29777
**Factor 7**	0.788682	4.92926
**Factor 8**	0.609656	3.81035
**Factor 9**	0.520457	3.25286
**Factor 10**	0.437123	2.73202
**Factor 11**	0.367110	2.29444
**Factor 12**	0.310372	1.93982
**Factor 13**	0.255101	1.59438
**Factor 14**	0.202614	1.26634
**Factor 15**	0.144765	0.90478
**Factor 16**	0.019503	0.12189
